# Medical school class presidents generate educational solutions during a global pandemic

**DOI:** 10.5116/ijme.60b8.a38b

**Published:** 2021-06-25

**Authors:** Jonathan Light, Aileen Lee, Teresa Elmore, Fergui Hernandez, Carrie Elzie

**Affiliations:** 1 School of Medicine, Eastern Virginia Medical School, Norfolk, VA, USA; 2 University of Arizona College of Medicine - Tucson, Tucson, AZ, USA; 3 School of Medicine, Virginia Commonwealth University, Richmond, VA, USA; 4 Keck School of Medicine of the University of Southern California, Los Angeles, CA, USA; 5 Department of Pathology and Anatomy, Eastern Virginia Medical School, Norfolk, VA, USA

**Keywords:** Student Leaders, Medical school class presidents, educational solutions, global pandemic, COVID-19

## Introduction

With roughly 83.56 million confirmed cases and 1.82 million confirmed deaths in 2020,^1^ COVID-19 has impacted ways of life worldwide, including transforming the educational landscape. Multiple stakeholders have been involved in managing the educational response to COVID-19, including executive boards and faculty. Less explored is the role of student leadership and its influences on an institution’s response to COVID-19.

One example of student leadership in the United States medical education context is that of class president. Typically, the medical school class president, elected by their peers, helps lead the Student Government Association as an inductee and is responsible for ensuring class leadership functionality and efficiency while serving as a liaison between students and the administration. As the chief class officer, the class president sets the agenda and establishes the order of business for the medical student class Executive Council (vice president, secretary, treasurer) and meetings. Furthermore, the class president is the conduit to medical school administration regarding student ideas and feedback, which was pivotal throughout the many changes this last year with COVID-19. In addition to these leadership roles, other traditional medical student-leader roles include curricular, extra-curricular, information technology, and Honor Council representations. Given the importance of medical school student leadership and the key role of the class president, it is important to understand how class presidents adapted and coordinated the institutional responses to COVID-19.

In a focus group, four medical school class presidents discussed the impact of the pandemic on the learning environment, identified potential solutions and shared their experiences. These experiences were recorded, transcribed, and analyzed for themes. The themes that emerged were on the roles of student leaders in communication, advocacy, community building and adaptation to this pandemic.

### Role of Student Leaders in Communication

A major impact of the pandemic was the sheer increase in the amount and type of necessary communication during times of isolation. A second-year medical school class president responsible for answering her class’s concerns while preparing for their United States Medical Licensing Examination Step 1, end of year assessment, explained that the “Frequency of communication changed as well with it becoming extraordinarily more difficult to keep in touch with Deans and individual students during isolation.” – Participant One. To adjust, class presidents needed to generate solutions to the overwhelming communication demands they encountered. For instance, the class presidents helped coordinate emails from the teachers and medical school administration to the students one week before exams rather than days before to reduce student burnout and maximize response rate. The role of the class president in the coordination of communication was further supported at the medical school level, according to one of the focus group participants. A survey conducted at this institution suggested that collaboration between the class president and institutional administration was seen as beneficial by more than 94% of respondents.

Leveraging technology also proved to be an effective strategy to manage communication demands. Group chats were encouraged by each of the class presidents in this focus group to enable students to efficiently connect with each other and other student leaders regarding educational matters. By implementing this solution, the medical school class presidents became conduits for their peers to build cohesion and exchange ideas.

### Role of Student Leaders in Advocacy

Medical school class presidents play a key role in advocating for their peers. The theme of “class advocate” arose in multiple domains across the four schools. Medical students are critical stakeholders in their education, and having their voice heard is of high importance in professional school programs.^2^ Traditionally, this is outward advocacy, as a representative of student concerns to stakeholders in administration. For example, class presidents can advocate for the perspective of their peers with faculty and administrators, but the pandemic made this more difficult because of the lack of proximity and direct communication. During the pandemic, this raised internal tensions for medical school class presidents. Bandwidth was an issue. Class presidents in the focus group reported an insurmountable number of emails detailing greater concerns from the student body around their education. Both the frequency and urgency of communication necessitated an almost constant availability of the class president. The increased demand for organizational and communication skills, in addition to an already demanding academic schedule, was challenging. One first-year medical school class president said, “I managed hundreds of informal and email requests from students concerning the curriculum, distance education, and virtual learning engagement ideas due to COVID-19 despite our medical school providing additional resources” - Participant Two.

### Role of Student Leaders in Class Community

The third major issue in which class presidents helped shape the institutional response to COVID-19 was in expanding the sense of community. At the beginning of the pandemic, there was already limited interaction and connection with peers, according to the focus group participants. This situation was exacerbated by the physical separation required with the transition to virtual learning. The pandemic threatened the ability of medical school class leaders to connect with their classmates and communicate their interest in securing leadership in future academic years. Further, new candidates seeking an elected position within their medical school class were challenged to introduce themselves to their school community. Identifying this problem necessitated the need to create a more engaging and inclusive platform for student leadership elections within a virtual environment. One medical school class president innovated the election process using a virtual election with a video speech platform. Compared to the written speech platform used pre-pandemic, candidates expressed positive feedback on the video process platform in establishing a social connection in a post-election survey that was distributed to the class. The other focus group participants developed a live virtual election process due to the pandemic.

The transformation in the election is just one example of a process change that stemmed from a desperate need to connect students while socially distanced. Class president focus group participants expressed the use of virtual learning environments to enhance inclusiveness and provide opportunities for student leaders to better serve their class throughout their distanced education experiences using a shared decision-making model. While technologies cannot replace the connectivity and relationships established through in-person flipped classrooms and clinical skills training in medical school, the disruption in educational processes due to COVID-19 led to innovations to help bridge student leadership with their constituents.

### The Importance of Role Clarity for Student Leaders

Role ambiguity has historically been a challenge of medical student representations^3^ and the consequences of the ambiguity became strained during the pandemic. One medical school class president in this focus group used a transformational leadership model^4^ under their medical school administration’s mentorship. The application of the transformational model helped clarify and redefine the roles of each medical school class leader, and this role clarity helped each leader transcend their personal interests in order to achieve the goals of the class.^5^ This participant shared that when defining student-leader roles, skills of consensus building, negotiation of responsibilities, and facilitated group discussion were required and leveraged diverse student perspectives. Further, decreasing role ambiguity helped increase task identity, expanded peer-to-peer support, promoted wellness, and decreased leadership burnout amidst the strain of the pandemic.

## Conclusions

This pandemic created a consistent need for student leaders to exercise crisis management skills which involved rapid response strategies.^6^ The class presidents expressed they remained flexible and felt better prepared to manage future problems given their experiences in the pandemic. Student leadership in medical school under the stress of the pandemic has helped prepare these focus group participants who served as class presidents to operate in the dynamic wards or clinics in which they will practice. Embracing a shared leadership paradigm^6^ at one medical school helped change task and communication distribution. A better alliance between medical school students and class presidents and between class presidents and medical school administration helped improve the learning environment. These partnerships, and the solutions identified by class presidents, were particularly important for underrepresented and economically disadvantaged students, who faced new barriers to medical education and were particularly impacted by the COVID-19 pandemic.^7^

In addition to the lessons learned by the medical school class presidents in their leadership through the pandemic, it is suggested that class leaders and institutions continue to use some practices even after the pandemic subsides. Clarified roles, use of the class president as a conduit for streamlined communication, improved community among peers, and reliance on virtual support environments are all mechanisms which had to be adopted due to the context of the pandemic and to protect ongoing student wellness. Because burnout in medical school is a substantial concern, all mechanisms adopted which resulted in a better community, student engagement, and mentorship by the administration should be cemented for future academic years. These practices that helped to promote student wellness and heightened academic support should outlast this global pandemic.

### Conflict of Interest

The authors declare that they have no conflict of interest.
